# Preoperative CT image analysis to improve risk stratification for clinically relevant pancreatic fistula after distal pancreatectomy

**DOI:** 10.1093/bjs/znac348

**Published:** 2022-10-29

**Authors:** Nicolò Pecorelli, Diego Palumbo, Giovanni Guarneri, Chiara Gritti, Francesco Prato, Marco Schiavo Lena, Alessia Vallorani, Stefano Partelli, Stefano Crippa, Claudio Doglioni, Francesco De Cobelli, Massimo Falconi

**Affiliations:** Division of Pancreatic Surgery, Pancreas Translational & Clinical Research Center, San Raffaele Scientific Institute, Milan, Italy; Faculty of Medicine, Vita-Salute San Raffaele University, Milan, Italy; Faculty of Medicine, Vita-Salute San Raffaele University, Milan, Italy; Department of Radiology, San Raffaele Scientific Institute, Milan, Italy; Division of Pancreatic Surgery, Pancreas Translational & Clinical Research Center, San Raffaele Scientific Institute, Milan, Italy; Faculty of Medicine, Vita-Salute San Raffaele University, Milan, Italy; Faculty of Medicine, Vita-Salute San Raffaele University, Milan, Italy; Department of Pathology, San Raffaele Scientific Institute, Milan, Italy; Faculty of Medicine, Vita-Salute San Raffaele University, Milan, Italy; Division of Pancreatic Surgery, Pancreas Translational & Clinical Research Center, San Raffaele Scientific Institute, Milan, Italy; Faculty of Medicine, Vita-Salute San Raffaele University, Milan, Italy; Division of Pancreatic Surgery, Pancreas Translational & Clinical Research Center, San Raffaele Scientific Institute, Milan, Italy; Faculty of Medicine, Vita-Salute San Raffaele University, Milan, Italy; Faculty of Medicine, Vita-Salute San Raffaele University, Milan, Italy; Department of Pathology, San Raffaele Scientific Institute, Milan, Italy; Faculty of Medicine, Vita-Salute San Raffaele University, Milan, Italy; Department of Radiology, San Raffaele Scientific Institute, Milan, Italy; Division of Pancreatic Surgery, Pancreas Translational & Clinical Research Center, San Raffaele Scientific Institute, Milan, Italy; Faculty of Medicine, Vita-Salute San Raffaele University, Milan, Italy

## Introduction

Clinically relevant postoperative pancreatic fistula (CR-POPF) still occurs in about 15 to 40 per cent patients who undergo distal pancreatectomy^1–4^. Improving risk stratification by identifying valid and reliable risk factors for CR-POPF after distal pancreatectomy may allow for a better understanding of possible mechanisms leading to pancreatic leak, assessment of the effectiveness of available mitigation strategies, and guide the development of novel interventions, with the ultimate goal of decreasing morbidity. Unfortunately, effective risk-stratification models based on readily available parameters such as the Fistula Risk Score (FRS)^5^ for pancreaticoduodenectomy are still unavailable for distal pancreatectomy^6^. The recently published distal pancreatectomy FRS (D-FRS) is still not used in clinical practice and includes variables that are heavily influenced by subjectivity, individual practice (i.e. operative time), and are difficult to interpret during minimally invasive distal pancreatectomy (i.e. pancreatic texture)^7^.

Preoperative CT has the potential to identify objectively both patient (i.e. anthropometric measures) and pancreatic-specific factors (i.e. gland size and characteristics) that may influence CR-POPF occurrence^8,9^, but only data from small retrospective series are available in patients undergoing distal pancreatectomy^10–12^. Therefore, the aim of this study was to determine the extent to which preoperative CT image analysis can improve risk stratification for CR-POPF after distal pancreatectomy.

## Results

**Fig. 1:**
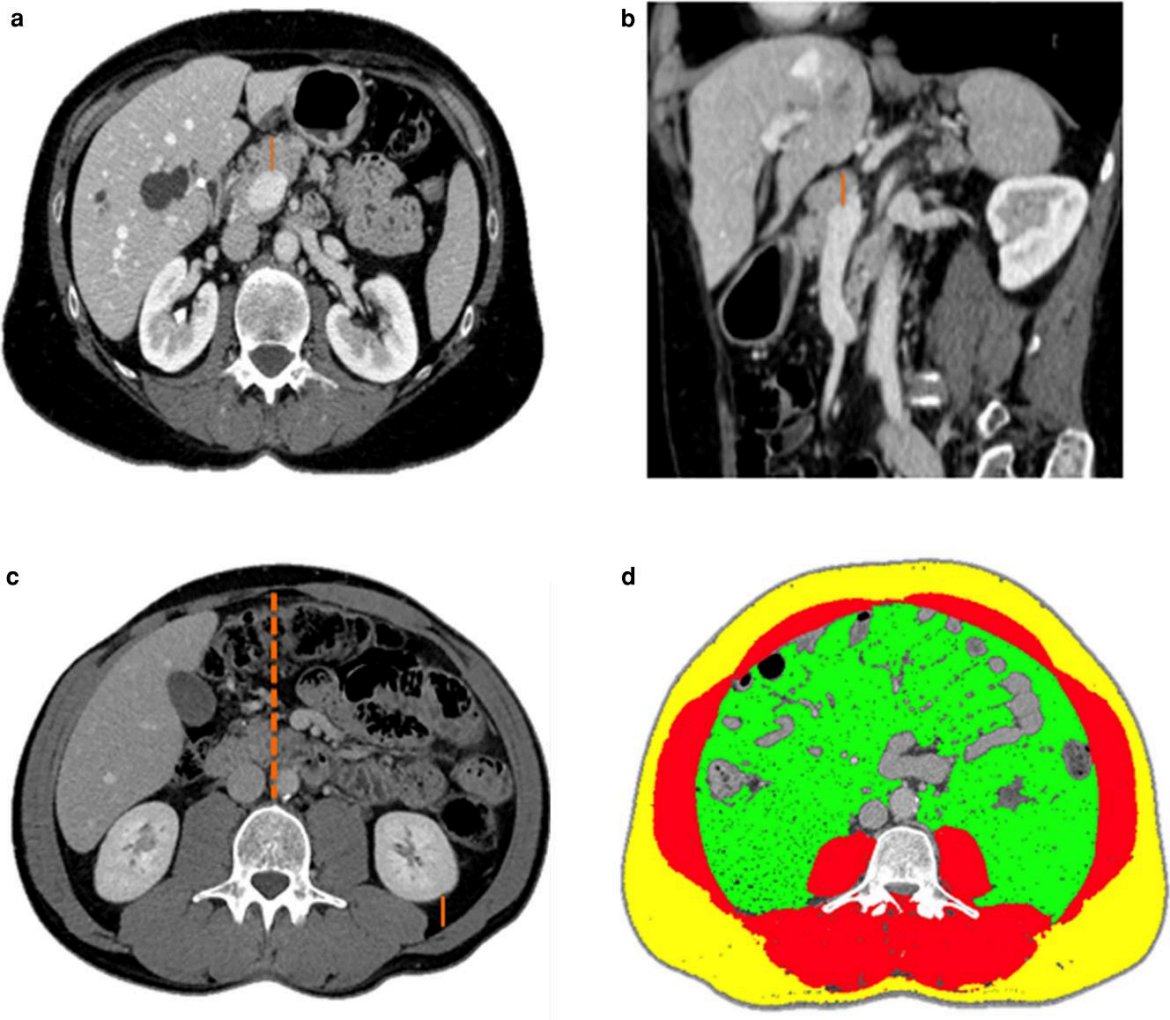
Assessment of quantitative CT parameters **a** On axial CT images, the anteroposterior (minor) pancreatic parenchymal thickness (orange line) was measured at the level of the splenomesenteric venous confluence. **b** The pancreatic parenchymal craniocaudal (major) thickness was identified on sagittal images along the maximum length of the gland. **c** On axial CT images, perirenal fat thickness (continuous orange line) was measured as the anteroposterior distance between the left posterior renal capsule and the adjacent junction of the posterior abdominal wall and corresponding paraspinal musculature. Intra-abdominal fat thickness (orange dashed line) was defined as the anteroposterior distance between the linea alba and the anterior surface of the third lumbar vertebra body. **d** Image postprocessing for the assessment of body composition parameters on axial CT at the level of the third lumbar vertebra. The subcutaneous fat area is highlighted in yellow, total abdominal muscle area in red, and visceral fat area in green.

Detailed methods are available in the *supplementary methods* and in *Appendix S1*. Data for 476 consecutive adult patients who underwent distal pancreatectomy between 2016 and 2021 at the Division of Pancreatic Surgery, San Raffaele Hospital (Milan, Italy) were retrospectively reviewed. CT imaging was unavailable for 156 (32.8 per cent) patients, who were then excluded from the study (*Table S1*). Included patients who underwent CT imaging analysis (*Fig. 1*, *Video 1, and Video 2*) were divided into a developmental cohort of 220 patients (2016 to 2019) and a validation cohort of 100 patients (2020 to 2021). The perioperative variables of the two cohorts are reported in *Table S2*.

In the developmental cohort, 74 patients (33.6 per cent) developed a CR-POPF 90 days postoperatively. Univariate analysis for factors associated with CR-POPF are summarized in *Table 1*. Clinical variables associated with CR-POPF were male sex, a history of coronary artery disease (CAD), a high BMI, and higher intraoperative blood loss. A thicker pancreas in both major (craniocaudal) and minor (anteroposterior) diameters was associated with CR-POPF, while pancreatic parenchymal attenuation and duct diameter had no effect. Patients with CR-POPF had a significantly higher intra-abdominal fat thickness, visceral fat area, and volume. A moderate correlation between radiological and pathological pancreatic measures was found (*Fig. S1*). *Table S3* shows the performance of radiological parameters as predictors of CR-POPF in the developmental cohort, and the ideal cut-off values identified for each variable.

**Table 1:** Univariate analysis for clinical and radiological predictors of clinically relevant postoperative pancreatic fistula (CR-POPF) in the developmental cohort (*n* = 220)

Variables	No CR-POPF (*n* = 146)	CR-POPF (*n* = 74)	*P* value
**Patient characteristics**			
Age (years)	65 (52–72)	63 (53–72)	0.532
Male sex	61 (41.8)	40 (54.1)	0.084
BMI (kg/m^2^)	23.51 (21.32–25.75)	25.50 (22.86–27.68)	0.004
BMI ≥ 25 kg/m^2^	52 (35.6)	41 (55.4)	0.005
ASA score ≥3	48 (32.9)	18 (24.3)	0.191
History of DM	33 (22.6)	11 (14.9)	0.175
History of CAD	8 (5.5)	9 (12.2)	0.079
History of chronic pulmonary disease	9 (6.2)	6 (8.1)	0.589
Preoperative chemotherapy	38 (26.0)	15 (20.3)	0.345
**Type of disease**			0.663
PDAC	64 (43.8)	33 (44.6)	
Neuroendocrine tumour	38 (26.0)	19 (25.7)	
IPMN	10 (6.8)	6 (8.1)	
Cystic neoplasms	19 (13.0)	8 (10.8)	
Other	15 (10.3)	8 (10.8)	
**Procedural factors**			
Successful laparoscopic resection	77 (52.7)	49 (66.2)	0.056
Spleen-preserving procedures	8 (5.5)	3 (4.1)	0.647
Associated major visceral resection	13 (8.9)	3 (4.1)	0.191
Vascular resection	8 (5.5)	3 (4.1)	0.647
Duration of surgery (min)	215 (174–249)	207 (166–248)	0.228
Estimated blood loss (ml)	200 (100–300)	200 (150–400)	0.676
Intraoperative fluid infusion (ml)	2200 (1525–2750)	2100 (1800–2875)	0.035
Intraoperative blood transfusion	5 (3.4)	0 (0)	0.107
**Radiological features**			
Main pancreatic duct diameter ≥3 (mm)	39 (26.7)	20 (27.0)	0.960
Pancreatic neck major diameter (mm)	28 (23–32)	30 (28–35)	<0.001
Pancreatic neck minor diameter (mm)	12 (9–14)	14 (11–16)	0.001
Predicted pancreatic neck area (mm^2^)	245.0 (188.5–339.7)	339.3 (233.3–400.6)	<0.001
Pancreatic fat oedema	33 (22.6)	18 (24.3)	0.775
L/E phase ratio pancreatic parenchyma	0.704 (0.561–0.903)	0.674 (0.532–0.941)	0.404
Liver density (HU)	58 (53–62)	57 (52–60)	0.140
TAMA (cm^2^/m^2^)	42.0 (37.4–48.2)	44.0 (38.1–50.1)	0.062
TMV (cm^3^)	32.3 (25.9–38.2)	34.9 (28.0–44.5)	0.008
VFA (cm^2^)	79.7 (29.9–153.2)	118.3 (60.5–194.0)	0.011
VFV (cm^3^)	18.4 (6.7–40.2)	32.1 (16.2–54.4)	0.004
SFA (cm^2^)	144.8 (109.8–202.5)	165.1 (125.3–213.4)	0.277
SFV (cm^3^)	38.81 (25.9–56.9)	42.6 (34.1–62.4)	0.068
VFA/TAMA ratio	1.71 (0.68–3.27)	2.63 (1.42–4.09)	0.017
Perirenal fat thickness (mm)	9.50 (5–16)	11 (5–21)	0.212
Intra-abdominal fat thickness (mm)	88 (71–108)	97 (80–120)	0.003

Values are expressed as n (%) or median (interquartile range). DM, diabetes mellitus; CAD, coronary artery disease; PDAC, Pancreatic ductal adenocarcinoma; IPMN, intraductal papillary mucinous neoplasm; L/E, late/early; HU, Hounsfield units; TAMA, total abdominal muscle area; TMV, total muscle volume; VFA, visceral fat area; VFV, visceral fat volume; SFA, superficial fat area; SFV, superficial fat volume.

Multivariate logistic regression models for factors associated with CR-POPF are provided in *Table 2*. Model A focused exclusively on clinical factors, including a BMI of 25 kg/m^2^ or higher and history of CAD, as parameters associated with CR-POPF. Model B, with simple radiological variables (i.e. without image postprocessing), included a BMI of 25 kg/m^2^ or higher as the only clinical variable, and both major and minor CT diameters of the pancreatic neck. In model C, with advanced radiological variables, the predicted pancreatic neck area and visceral fat volume were independent risk factors for CR-POPF. The area under the receiver operating curve (AUC) for model A in predicting CR-POPF was 0.651 (95 per cent c.i. 0.584 to 0.734; *P* < 0.001). Radiological models B (AUC 0.725, 95 per cent c.i. 0.55 to 0.794; *P* < 0.001) and C (AUC 0.733, 95 per cent c.i. 0.664 to 0.801; *P* < 0.001) demonstrated better accuracy. *Table S4* and *Fig. S2* show multivariate models B and C, and ROC curves using ideal cut-offs for continuous radiological variables in the developmental cohort.

**Table 2:** Multivariate logistic regression models for clinical and radiological variables associated with clinically relevant postoperative pancreatic fistula (CR-POPF) in the developmental cohort (*n* = 220)

	Beta coefficient	OR	95% c.i.	*P* value
**Model A (only clinical variables model)** AUC 0.651, 95% c.i. 0.58–0.73, *P* < 0.001				
BMI ≥25 kg/m^2^	0.888	2.429	1.34–4.39	0.003
ASA > 3	−0.618	0.539	0.26–1.12	0.097
Diabetes	−0.709	0.492	0.21–1.14	0.100
CAD	1.479	4.390	1.36–14.19	0.013
**Model B (simplified radiological model**) AUC 0.725, 95% c.i. 0.66–0.79, *P* < 0.001				
BMI ≥25 kg/m^2^	0.835	2.305	1.23–4.31	0.009
Intraoperative blood loss >200 ml	0.635	1.887	0.97–3.67	0.061
Pancreatic neck major diameter*	0.088	1.092	1.04–1.15	0.001
Pancreatic neck minor diameter*	0.116	1.123	1.02–1.23	0.017
**Model C (advanced radiological model)** AUC 0.733, 95% c.i. 0.64–0.80, *P* < 0.001				
ASA score ≥3	−0.877	0.416	0.18–1.00	0.050
Intraoperative blood loss >200 ml	0.657	1.929	0.94–3.97	0.074
Predicted pancreatic neck area†	0.052	1.053	1.02–1.09	0.002
VFV‡	0.098	1.102	1.03–1.18	0.006

Radiological diameters of the pancreatic neck are expressed as continuous variables. Odds ratio (OR) refers to each increase of 1 mm in diameter. †Radiologically calculated pancreatic remnant stump area is expressed as a continuous variable. OR refers to each increase of 10 mm^2^of the area. ‡Visceral fat volume (VFV) is expressed as a continuous variable. OR refers to each increase of 5 cm^3^ in visceral fat volume. AUC, area under the receiver operating curve; CAD, coronary artery disease.

In the validation cohort, the observed risk for CR-POPF was 32 per cent. The predicted mean risk was 46 per cent for model A, 23 per cent for model B, and 25 per cent for model C. The predicted and observed risks in models B and C were similar, according to the Hosmer–Lemeshow test (*P* = 0.154 and *P* = 0.207, respectively), while model A demonstrated lower accuracy (*P* = 0.059). ROC curves showing the discriminative power of the three models in the developmental and validation cohort are provided in *Fig. S3*.

## Discussion

In the present study, which included a large cohort of consecutive distal pancreatectomies performed at a high-volume centre, CT assessment of anthropometric and pancreatic gland measures yielded predictive models with improved discriminative power *versus* only clinical variables. These findings suggest that it could be used in future research as a risk stratification tool to compare the effectiveness of perioperative prevention and mitigation strategies for CR-POPF.

Pancreatic parenchymal characteristics (e.g. thickness, texture, and duct dilation) may play an important role in the occurrence of CR-POPF^7,13^, but they are influenced by intraoperative subjective assessment, which is even more challenging during minimally invasive surgery, where haptic feedback is deficient. In the present study, we overcame this important limitation by performing a preoperative radiological systematic assessment of pancreatic characteristics, including gland thickness and adiposity. Notably, we found no association between pancreatic parenchymal low CT attenuation^14^, a sign of a ‘soft’, ‘fatty’ pancreas, and CR-POPF. Conversely, parenchymal thickness, measured either as a unidimensional diameter or bi-dimensional area at the pancreatic neck (i.e. the most common transection site for distal pancreatectomy), showed a significant association with CR-POPF. This finding suggests that, in addition to leaving a larger stump area, a thicker gland is also more difficult to manage and there is higher chance of crushing the pancreatic parenchyma and rupturing the pancreatic capsule when stapling or suturing. To overcome this technical challenge, Asbun *et al*. proposed a progressive stepwise compression technique and reinforced staple line for pancreatic transection, reporting CR-POPF rates of lower than 10 per cent, but other groups have failed to reproduce their outcomes^15^.

A unique feature of our research was the evaluation of body composition measures. In this study we found that a high visceral adiposity was the strongest factor correlating with CR-POPF, confirming the results of a smaller cohort study by Vanbrugghe *et al*.^12^. Visceral obesity appears to be a stronger parameter than BMI for risk stratification, and is considered the key component of metabolic syndrome^16^. It is associated with chronic inflammation and insulin resistance, which may explain its negative effects on surgical outcomes^17^.

A key issue is how our findings can improve patient management. Preoperatively, more precise risk stratification can help the surgeon counsel the patient and their family regarding the actual risks of developing CR-POPF. It is still unclear if viscerally obese patients can benefit from tailored nutritional and physical prehabilitation^18^. Intraoperatively, CT prediction could allow for subjective surgeon judgement of a high-risk (that is thick and soft pancreatic stump) *versus* low-risk situation to be surpassed, and for a selective intraperitoneal drain placement strategy to be adopted, based on objective data *versus* ‘gut feeling’. Correlation of preoperative radiological pancreatic neck diameters with pathology measures of the resection margin in our series were valid, confirming the accuracy of CT for this purpose. Nonetheless, it would be interesting to evaluate, in a prospective fashion, the correlation between calculated preoperative risk and the surgeon’s intraoperative judgement. The two pieces of information may be complimentary in a Bayesian stepwise approach to CR-POPF prediction. Finally, external validation would be mandatory to verify the accuracy of our models in other pancreatic surgery centres.

## Supplementary Material

znac348_Supplementary_Data

## Data Availability

The data that support the findings of this study are available from the corresponding author, upon reasonable request.
