# Supplementary material for: Preoperative CT image analysis to improve risk stratification for clinically relevant pancreatic fistula after distal pancreatectomy
Source: Br J Surg. 2022 Oct 29;110(8):891–5. doi: 10.1093/bjs/znac348 (PMC10481779; doi:10.1093/bjs/znac348)
Supplement: znac348_Supplementary_Data [file znac348_Supplementary_Data.docx]

**Preoperative CT scan image analysis to improve risk stratification for clinically relevant pancreatic fistula after distal pancreatectomy**

Nicolò Pecorelli^1,2^, Diego Palumbo^2,3^, Giovanni Guarneri^1^, Chiara Gritti^2^, Francesco Prato^2^, Marco Schiavo Lena^4^, Alessia Vallorani^2^, Stefano Partelli^1,2^, Stefano Crippa^1,2^, Claudio Doglioni^2,4^, Francesco De Cobelli^2,3^, Massimo Falconi^1,2^.

^1^ Division of Pancreatic Surgery, Pancreas Translational & Clinical Research Center, San Raffaele Scientific Institute, Milan, Italy

^2^ Vita-Salute San Raffaele University, Milan, Italy

^3^ Department of Radiology, San Raffaele Scientific Institute, Milan, Italy

^4^ Department of Pathology, San Raffaele Scientific Institute, Milan, Italy

**Corresponding author:**

Massimo Falconi, MD

Division of Pancreatic Surgery, San Raffaele Scientific Institute

Via Olgettina 60, Milano 20132, Italy

[falconi.massimo@hsr.it](about:blank)

**Supplementary Materials - Index**

| **Supplementary Methods** | *pag. 2* |
| --- | --- |
| **Supplementary Appendix** |  |
| Appendix S1. STROBE checklist | *pag. 6* |
| **Supplementary Tables and Figures** |  |
| Table S1 | *pag. 9* |
| Table S2 | *pag. 11* |
| Table S3 | *pag. 13* |
| Table S4 | *pag. 14* |
| Figure S1 | *pag. 15* |
| Figure S2 | *pag. 16* |
| Figure S3 | *pag. 17* |
| **References** | *pag. 18* |

# Supplementary Methods

## Study design

This single center retrospective cohort study was conducted following the Strengthening for the Reporting of Observational Studies in Epidemiology Statement (STROBE) guidelines ^1^ (available as **Supplementary Appendix**). A formal ethical committee approval was waived due to the retrospective nature of the study, according to our institutional policy.

Consecutive adult patients who underwent DP at the Division of Pancreatic Surgery at San Raffaele Hospital from January 2016 to December 2019 were included in the developmental cohort of the study. The external validation set was composed of all patients who underwent DP at the same institution between January 2020 and May 2021.

## Surgical procedures and perioperative care

All procedures were carried out by a high-volume pancreatic surgery ^2^ team. Pancreatic transection was performed with a scalpel followed by pancreatic duct selective suturing during open surgery and by stapler closure during laparoscopy.

All patients were managed following an established perioperative care enhanced recovery after surgery pathway, as previously described ^3^. One flat Penrose drain (12 mm; Redax^®^) was placed in proximity of the pancreatic stump and managed according to drain fluid amylase concentration on POD 5 as previously reported ^4^.

## Radiological image analysis

Preoperative contrast-enhanced multiphase CT scan imaging performed within 30 days before index surgery was retrieved form digital storage system and provided for radiological review. Three readers (F.P., C.G., D.P.) with different experience in abdominal CT independently analyzed all images blinded to patient postoperative outcomes. Specifically, a medical school intern (6 months-experience), a radiology resident (3 years-experience) and a board-certified radiologist (10 years-experience) were involved.

Two sets of quantitative CT parameters were selected for analysis: future pancreatic remnant measurements [i.e., pancreatic thickness, main pancreatic duct (MPD) diameter, late phase/early phase (L/E) ratio] and body composition parameters [i.e., perirenal fat (PRF) and intra-abdominal fat (IAF) thickness, total abdominal muscle area (TAMA) and volume (TMV), visceral fat area (VFA) and volume (VFV), subcutaneous fat area (SFA) and volume (SFV)].

Pancreatic neck parenchymal thickness was systematically assessed on axial and parasagittal CT images as shown in **Figure 1a-b**. Predicted parenchymal neck surface area was calculated as an elliptical area [area = π*(anteroposterior diameter/2)*(craniocaudal diameter/2)]. Pancreatic texture was evaluated by L/E ratio systematically sampled two centimeters to the left of the spleno-mesenteric venous confluence as previously described ^5^. PRF and IAF thickness were measured on axial CT at the level of the third lumbar vertebra body (L3) as depicted in **Figure 1c** ^6^. In the video (**Video S1**) are shown measurements of PRF, IAF, MPD and pancreatic parenchymal diameters.

Image post-processing as shown in **Figure 1d** was carried out with commercially available software (sliceOmatic version 5.0 software, Tomovision®, Montreal, QC, Canada); further details about measurement of body composition parameters (TAMA, VFA, SFA) assessment have been previously published ^7^. TMV, SFV and VFV were automatically calculated computing the surface area of each tag value covered by a single image slice and then multiplying it by the image thickness^8^. The video (**Video S2**) illustrates image post-processing and delineation of different body composition areas.

## Pathological data analysis

Haematoxylin and eosin (H & E) stained sections (between 1 and 4 slides for each case) of the pancreatic resection margin were reviewed by 2 expert pancreatic pathologists (M.S.L., C.D.), who were blind to all clinical information. Main pancreatic duct diameter as measured at the pancreatic resection margin at the time of gross examination was reported. Major and minor axial diameters of the pancreatic stump were assessed as well.

## Definition of outcomes and data collection

The main study outcome was CR-POPF at 90 days after surgery, defined as grade B-C pancreatic fistula according to the 2016 classification of the International Study Group on Pancreatic Surgery (ISGPS) ^9^. Other pancreatic surgery specific postoperative outcomes were defined according to ISGPS consensus ^10,11^. Complication severity was measured by Dindo-Clavien classification and Complication Comprehensive Index (CCI) ^12,13^.

Data were collected from the institutional prospectively maintained electronic pancreatic surgery database.

## Statistical analysis

Continuous variables are presented as medians [interquartile range (IQR)] and were compared using Mann-Whitney U-test. Categorical variables are reported as frequencies (percentages) and were compared by Pearson chi-square test. Binary logistic regression analysis was conducted in order to assess risk factors for CR-POPF in the developmental cohort. Three multivariate models including parameters with increasing level of complexity were obtained through stepwise backward conditional elimination of the different variables included (Wald method, p ≤ 0.05 for entry, p> 0.10 for removal). Model A included only clinical variables, model B simple radiological features with no need for image post-processing, and model C advanced radiological measures. Results were expressed as Odds Ratio (OR) and 95% confidence interval (CI). A Receiver Operating Characteristic (ROC) curve analysis was computed to assess the predictive power of each model.

A predicted risk was calculated for every patient in the validation cohort based on regression coefficients from previous models. The predicted risk was tested in the validation population by C-statistics and the Hosmer-Lemeshow test. C-statistics was obtained by the area under the curve (AUC) of a ROC curve analysis testing predicted risk for CR-POPF.

Inter-observer reliability for CT scan parameters was evaluated by intraclass correlation coefficient (ICC). ICC values of 0.70 or higher indicate good reliability ^14^. Pearson’s correlation analysis was performed to assess the level of concordance between radiological and pathological measures of the pancreatic neck. Correlation coefficients between 0.4 and 0.69 indicate moderate correlation, values above 0.7 strong correlation ^15^.

A p< 0.050 was considered as statistically significant. All analyses were conducted using the Statistical Package for the Social Science (SPSS, Inc., Chicago, Il), version 25.

# Supplementary Appendixes

**Appendix S1. STROBE checklist**

|  | | Item No | Recommendation | Section – page number |
| --- | --- | --- | --- | --- |
| **Title and abstract** | | 1 | (*a*) Indicate the study’s design with a commonly used term in the title or the abstract | Supplementary material |
|  |  |  | (*b*) Provide in the abstract an informative and balanced summary of what was done and what was found | Not applicable |
| Introduction | | | |  |
| Background/rationale | | 2 | Explain the scientific background and rationale for the investigation being reported | Introduction Pag. 2 |
| Objectives | | 3 | State specific objectives, including any prespecified hypotheses | Introduction Pag. 2 |
| Methods | | | |  |
| Study design | | 4 | Present key elements of study design early in the paper | Supplementary methods - study design Pag 2 |
| Setting | | 5 | Describe the setting, locations, and relevant dates, including periods of recruitment, exposure, follow-up, and data collection | Supplementary methods - study design Pag 2 |
| Participants | | 6 | (*a*) *Cohort study*—Give the eligibility criteria, and the sources and methods of selection of participants. Describe methods of follow-up  *Case-control study*—Give the eligibility criteria, and the sources and methods of case ascertainment and control selection. Give the rationale for the choice of cases and controls  *Cross-sectional study*—Give the eligibility criteria, and the sources and methods of selection of participants | Supplementary methods Pag 2-3 |
|  |  |  | (*b*) *Cohort study*—For matched studies, give matching criteria and number of exposed and unexposed  *Case-control study*—For matched studies, give matching criteria and the number of controls per case | Supplementary methods - Pag 2-3  Table S1 and S2 |
| Variables | | 7 | Clearly define all outcomes, exposures, predictors, potential confounders, and effect modifiers. Give diagnostic criteria, if applicable | Supplementary methods - Definition of outcomes and data collection Pag. 4 |
| Data sources/ measurement | | 8* | For each variable of interest, give sources of data and details of methods of assessment (measurement). Describe comparability of assessment methods if there is more than one group | Supplementary methods- Radiological image analysis Pag. 2-3 |
| Bias | | 9 | Describe any efforts to address potential sources of bias | Supplementary methods  Pag 3-4 |
| Study size | | 10 | Explain how the study size was arrived at | Supplementary methods  Pag 3-4 |
| Quantitative variables | | 11 | Explain how quantitative variables were handled in the analyses. If applicable, describe which groupings were chosen and why | Supplementary methods  - Definition of outcomes and data collection Pag. 4 |
| Statistical methods | | 12 | (*a*) Describe all statistical methods, including those used to control for confounding | Supplementary methods – Statistical analysis Pag. 4-5 |
|  |  |  | (*b*) Describe any methods used to examine subgroups and interactions | Supplementary methods – Statistical analysis Pag. 4-5 |
|  |  |  | (*c*) Explain how missing data were addressed | Supplementary methods – Statistical analysis Pag. 4-5 |
|  |  |  | (*d*) *Cohort study*—If applicable, explain how loss to follow-up was addressed  *Case-control study*—If applicable, explain how matching of cases and controls was addressed  *Cross-sectional study*—If applicable, describe analytical methods taking account of sampling strategy | Supplementary methods – Statistical analysis Pag. 4-5 |
|  |  |  | (*e*) Describe any sensitivity analyses |  |
| Results | | | |  |
| Participants | 13* | (a) Report numbers of individuals at each stage of study—e.g- numbers potentially eligible, examined for eligibility, confirmed eligible, included in the study, completing follow-up, and analyzed | | Results Pag. 2-3 |
|  |  | (b) Give reasons for non-participation at each stage | | Results Pag. 2-3 |
|  |  | (c) Consider use of a flow diagram | | n.a. |
| Descriptive data | 14* | (a) Give characteristics of study participants (e.g. demographic, clinical, social) and information on exposures and potential confounders | | Results Pag. 2-3  Table 1  Table S1 and S2 |
|  |  | (b) Indicate number of participants with missing data for each variable of interest | | Table 1  Table S1 and S2 |
|  |  | (c) *Cohort study*—Summarize follow-up time (e.g., average and total amount) | | n.a. |
| Outcome data | 15* | *Cohort study*—Report numbers of outcome events or summary measures over time | | Results Pag. 3-4  Table 1 |
|  |  | *Case-control study—*Report numbers in each exposure category, or summary measures of exposure | |  |
|  |  | *Cross-sectional study—*Report numbers of outcome events or summary measures | |  |
| Main results | 16 | (*a*) Give unadjusted estimates and, if applicable, confounder-adjusted estimates and their precision (e.g,, 95% confidence interval). Make clear which confounders were adjusted for and why they were included | | Results Pag. 3-4  Table 1-2 |
|  |  | (*b*) Report category boundaries when continuous variables were categorized | | Results Pag. 3-4  Table 2  Table S3 and S4 |
|  |  | (*c*) If relevant, consider translating estimates of relative risk into absolute risk for a meaningful time period | | Results Pag. 3-4  Table 2  Table S3 and S4 |
| Other analyses | 17 | Report other analyses done—e.g. analyses of subgroups and interactions, and sensitivity analyses | | Table S3 and S4  Figure S1-S2 |
| Discussion | | | |  |
| Key results | 18 | Summarize key results with reference to study objectives | | Discussion Pag. 4-5 |
| Limitations | 19 | Discuss limitations of the study, taking into account sources of potential bias or imprecision. Discuss both direction and magnitude of any potential bias | | Discussion Pag. 4-5 |
| Interpretation | 20 | Give a cautious overall interpretation of results considering objectives, limitations, multiplicity of analyses, results from similar studies, and other relevant evidence | | Discussion Pag. 4-5 |
| Generalizability | 21 | Discuss the generalizability (external validity) of the study results | | Discussion Pag. 4-5 |
| Other information | | | |  |
| Funding | 22 | Give the source of funding and the role of the funders for the present study and, if applicable, for the original study on which the present article is based | | Disclosure Pag. 1 |

# Supplementary Figures and Tables

**Table S1:** table reporting preoperative characteristics and perioperative outcomes according to the availability of adequate imaging

|  | **Developmental cohort** | **No CT imaging available** | **CT imaging analyzed** | **p-value** |
| --- | --- | --- | --- | --- |
| **Variables** | ***n= 376*** | ***n= 156*** | ***n= 220*** |  |
| Age (years), median [IQR] | 64 [52-71] | 62 [52-69] | 65 [53-73] | 0.034 |
| Male gender | 214 (56.9%) | 61 (39.1%) | 101 (45.9%) | 0.189 |
| BMI (kg/m^2^), median [IQR] | 24.49 [21.84-26.73] | 24.81 [21.91-27.05] | 24.16 [21.73-26.36] | 0.397 |
| *Comorbidities* |  |  |  |  |
| History of diabetes mellitus | 67 (17.8%) | 23 (14.7%) | 44 (20.0%) | 0.189 |
| History of coronary artery disease | 29 (7.7%) | 17 (7.7%) | 17 (7.7%) | 0.990 |
| History of pulmonary disease | 22 (5.9%) | 7 (4.5%) | 15 (6.8%) | 0.343 |
| ASA score ≥ 3 | 113 (30.1%) | 47 (30.1%) | 66 (53.2%) | 0.979 |
| Preoperative chemotherapy | 78 (20.7%) | 25 (16.0%) | 53 (24.1%) | 0.057 |
| *Type of disease* |  |  |  | 0.007 |
| PDAC | 144 (38.3%) | 47 (30.1%) | 97 (44.1%) |  |
| Neuroendocrine tumor | 95 (25.3%) | 38 (24.4%) | 57 (25.9%) |  |
| IPMN | 39 (10.4%) | 23 (14.7%) | 16 (7.3%) |  |
| Cystic neoplasms | 52 (13.8%) | 29 (18.6%) | 23 (10.5%) |  |
| Other | 46 (12.2%) | 19 (12.2%) | 27 (12.3%) |  |
| Successful laparoscopy | 226 (60.1%) | 100 (64.1%) | 126 (57.3%) | 0.183 |
| Spleen preserving procedures | 24 (6.4%) | 13 (8.3%) | 11 (5.0%) | 0.193 |
| Associated major visceral resection | 24 (6.4%) | 8 (5.1%) | 16 (7.3%) | 0.402 |
| Vascular resection | 16 (4.3%) | 5 (3.2%) | 11 (5.0%) | 0.396 |
| Duration of surgery (minutes), median [IQR] | 213 [172-250] | 210 [172-252] | 215 [173-248] | 0.781 |
| Blood loss (ml), median [IQR] | 200 [100-300] | 200 [100-300] | 200 [100-300] | 0.759 |
| Intraoperative fluid infusion (ml), median [IQR] | 2200 [1625-2800] | 2250 [1600-2800] | 2200 [1700-2800] | 0.752 |
| Intraoperative blood transfusion | 13 (3.5%) | 8 (5.1%) | 5 (2.3%) | 0.135 |
| LOS, median [IQR] | 7 [6-9] | 7 [6-10] | 6 [6-8] | <0.001 |
| TFR. Median [IQR] | 5 [4-7] | 6 [5-7] | 5 [4-6] | <0.001 |
| Overall complications | 255 (67.8%) | 104 (66.7%) | 151 (68.6%) | 0.687 |
| Major complications | 58 (15.4%) | 27 (17.3%) | 31 (14.1%) | 0.395 |
| Postoperative mortality | 2 (0.5%) | 2 (1.3%) | 0 (0%) | 0.092 |
| CR-POPF | 129 (34.3%) | 55 (35.3%) | 74 (33.6%) | 0.744 |
| DGE | 12 (3.2%) | 6 (3.8%) | 6 (2.7%) | 0.543 |
| PPH | 19 (5.1%) | 8 (5.1%) | 11 (5.0%) | 0.955 |
| SSI | 49 (13.1%) | 22 (14.1%) | 27 (12.3%) | 0.604 |
| CCI, mean (95%CI) | 15.43 (13.76-17.10) | 16.47 (13.60-19.34) | 14 .69 (12.67-16.71) | 0.424 |

BMI: body mass index; ASA; America society of anaesthesiology; PDAC; pancreatic ductal adenocarcinoma; IPMN; intraductal papillary mucinous neoplasm; LOS: length of hospital stay; TFR: time to functional recovery; CR-POPF: clinically-relevant post-operative pancreatic fistula; DGE: delayed emptying^10^; PPH: post-pancreatectomy haemorrhage^11^; CCI: comprehensive complication index^13^; SSI: surgical-site infections

**Table S2:** preoperative characteristics and perioperative outcomes according to the availability of adequate imaging

|  | **Developmental cohort with available CT** | **Validation**  **cohort** | **p-value** |
| --- | --- | --- | --- |
| **Variables** | ***n= 220*** | ***n= 100*** |  |
| Age (years), median [IQR] | 65 [53-73] | 64 [57-73] | 0.652 |
| Male gender | 101 (45.9%) | 50 (50.0%) | 0.497 |
| BMI (kg/m^2^), median [IQR] | 24.16 [21.73-26.36] | 24.96 [22.23-28.09] | 0.083 |
| BMI ≥ 25 kg/m^2^ | 93 (42.3%) | 50 (50.0%) | 0.197 |
| *Comorbidities* |  |  |  |
| History of diabetes mellitus | 44 (20.0%) | 16 (16.0%) | 0.395 |
| History of coronary artery disease | 17 (7.7%) | 6 (6.0%) | 0.579 |
| History of pulmonary disease | 15 (6.8%) | 7 (7.0%) | 0.952 |
| ASA score ≥3 | 66 (30.0%) | 30 (30.0%) | 0.857 |
| Preoperative chemotherapy | 53 (24.1%) | 28 (28.0%) | 0.456 |
| *Type of disease* |  |  | 0.417 |
| PDAC | 97 (44.1%) | 42 (42.0%) |  |
| Neuroendocrine tumor | 57 (25.9%) | 24 (24.0%) |  |
| IPMN | 16 (7.3%) | 10 (10.0%) |  |
| Benign cystic neoplasms | 23 (10.4%) | 11 (11.0%) |  |
| Other | 27 (12.3%) | 13 (13.0%) |  |
| Successful laparoscopy | 126 (57.3%) | 51 (51.0%) | 0.295 |
| Spleen preserving procedures | 11 (5.0%) | 6 (6.0%) | 0.712 |
| Associated major visceral resection | 16 (7.3%) | 9^‡^ (9.0%) | 0.594 |
| Vascular resection | 11 (5.0%) | 4 (4.0%) | 0.695 |
| Duration of surgery (minutes), median [IQR] | 213 [171-248] | 214 [177-270] | 0.121 |
| Blood loss (ml), median [IQR] | 200 [100-300] | 200 [100-350] | 0.199 |
| Intraoperative fluid infusion (ml), median [IQR] | 2200 [1700-2800] | 3000 [2000-3700] | 0.001 |
| Intraoperative blood transfusion | 5 (2.3%) | 4 (4.0%) | 0.386 |
| LOS, median [IQR] | 6 [6-8] | 7 [5-8] | 0.779 |
| No complications | 69 (31.4%) | 41 (41.0%) | 0.093 |
| Minor complications at 90 days^12^ |  |  | 0.010 |
| Dindo-Clavien I | 58 (26.4%) | 14 (14.0%) |  |
| Dindo-Clavien II | 62 (28.2%) | 22 (22.0%) |  |
| Major complications at 90 days^12^ |  |  | 0.049 |
| Dindo-Clavien IIIa | 21 (9.5%) | 20 (20.0%) |  |
| Dindo-Clavien IIIb | 9 (4.1%) | 2 (2.0%) |  |
| Dindo-Clavien IV | 1 (0.5%) | 1 (1.0%) |  |
| Mortality | 0 (0%) | 1 (1.0%) | 0.137 |
| Clinically relevant pancreatic fistula | 74 (33.7%) | 32 (32.0%) | 0.773 |
| Grade B | 69 (31.4%) | 30 (30%) |  |
| Grade C | 5 (2.3%) | 2 (2%) |  |
| Delayed gastric emptying | 6 (2.7%) | 5 (5.0%) | 0.301 |
| Post-pancreatectomy hemorrhage | 11 (5.0%) | 2 (2.0%) | 0.208 |
| CCI, mean (95%CI) | 14.94 (12.87-17.01) | 15.31 (13.69-16.88) | 0.771 |

BMI: body mass index; ASA; America society of anaesthesiology; PDAC; pancreatic ductal adenocarcinoma; IPMN; intraductal papillary mucinous neoplasm; LOS: length of hospital stay; CCI: comprehensive complication index

⁑ 8 small bowel or colonic resections, 6 partial gastric resections, 4 liver resections, 1 nephrectomy, 5 other resections

‡ 3 small bowel or colic resections, 3 gastric resections, 2 liver resections, 1 other resection

**Table S3:** Area under the ROC curve (AUC) for radiological parameters as predictors of clinically relevant pancreatic fistula in the developmental cohort and ideal cut-offs identified for each variable in the developmental cohort.

|  |  |  |  | **Ideal cut-off performance** | | | | | |
| --- | --- | --- | --- | --- | --- | --- | --- | --- | --- |
| **Variables** | **AUC** | **95%CI** | **p** | **Cut-off** | **Sens** | **Spec** | **OR** | **95%CI** | **p** |
| Radiological Wirsung diameter, mm | 0.486 | 0.40-0.57 | 0.754 | n.a. |  |  |  |  |  |
| Pancreatic neck major diameter | 0.633 | 0.55-0.72 | 0.003 | ≥25mm | 81% | 60% | 2.805 | 1.36-5.81 | 0.005 |
| Pancreatic neck minor diameter | 0.610 | 0.53-0.69 | 0.014 | ≥10mm | 77% | 58% | 2.361 | 1.07-5.22 | 0.033 |
| Pancreatic neck area | 0.668 | 0.59-0.75 | <0.001 | ≥250 mm^2^ | 72% | 62% | 3.143 | 1.70-5.81 | <0.001 |
| TMV | 0.591 | 0.50-0.68 | 0.044 | <30cm^3^ | 65% | 56% | 1.651 | 0.93-2.95 | 0.087 |
| VFA | 0.614 | 0.53-0.70 | 0.011 | ≥60cm^2^ | 76% | 54% | 2.361 | 1.26-4.41 | 0.007 |
| VFV | 0.625 | 0.51-0.71 | 0.005 | ≥18 cm^3^ | 75% | 55% | 2.817 | 1.52-5.21 | 0.001 |
| SFA | 0.566 | 0.48-0.65 | 0.144 | n.a. |  |  |  |  |  |
| SFV | 0.588 | 0.50-0.67 | 0.050 | ≥40 cm^3^ | 62% | 52% | 1.426 | 0.81-2.51 | 0.218 |
| VFA/TAMA | 0.614 | 0.53-0.70 | 0.011 | ≥1 | 79% | 51% | 1.667 | 0.87-3.21 | 0.126 |
| Perirenal fat thickness | 0.558 | 0.47-0.65 | 0.193 | n.a. |  |  |  |  |  |
| Intra-abdominal fat thickness | 0.611 | 0.53-0.69 | 0.014 | ≥80 mm | 75% | 61% | 2.051 | 1.09-3.83 | 0.025 |

OR: Odds Ratio; Sens: sensitivity; Spec: specificity; n.a.; not available; TMV: total muscle volume; VFA: visceral fat area; VFV: visceral fat volume; SFA: superficial fat area; SFV: superficial fat volume; TAMA: total abdominal muscle area; n.a. not available.

**Table S4:** table reporting multivariate logistic regression models for factors associated with clinically relevant pancreatic fistula using ideal cut-offs for continuous radiological variables in the developmental cohort.

|  | **Beta coefficient** | **OR** | **C.I. 95%** | **p-value** |
| --- | --- | --- | --- | --- |
| *Model B: Radiological simplified model* |  |  |  |  |
| BMI ≥ 25 kg/m^2^ | 0.902 | 2.464 | 1.48-4.10 | 0.001 |
| Intraoperative blood loss > 200 ml | 0.740 | 2.066 | 1.25-3.52 | 0.005 |
| Radiological pancreatic neck major diameter ≥ 25 mm | 1.519 | 4.567 | 2.20-9.46 | <0.001 |
| Radiological pancreatic neck minor diameter ≥ 10 mm | 0.607 | 1.660 | 0.91-3.53 | 0.089 |
| *Model C: Radiological advanced model* |  |  |  |  |
| ASA score ≥3 | -0.597 | 0.551 | 0.31-0.99 | 0.046 |
| Intraoperative blood loss >200 ml | 0.661 | 1.936 | 1.12-3.23 | 0.012 |
| Radiological pancreatic neck area ≥250 mm^2^ | 0.989 | 2.689 | 1.53-4.73 | 0.001 |
| VFV ≥ 18 cm^3^ | 0.960 | 2.611 | 1.48-4.59 | 0.001 |

OR: Odds Ratio; BMI: body mass index; ASA: American society of Anesthesiologists; VFV: visceral fat volume

**
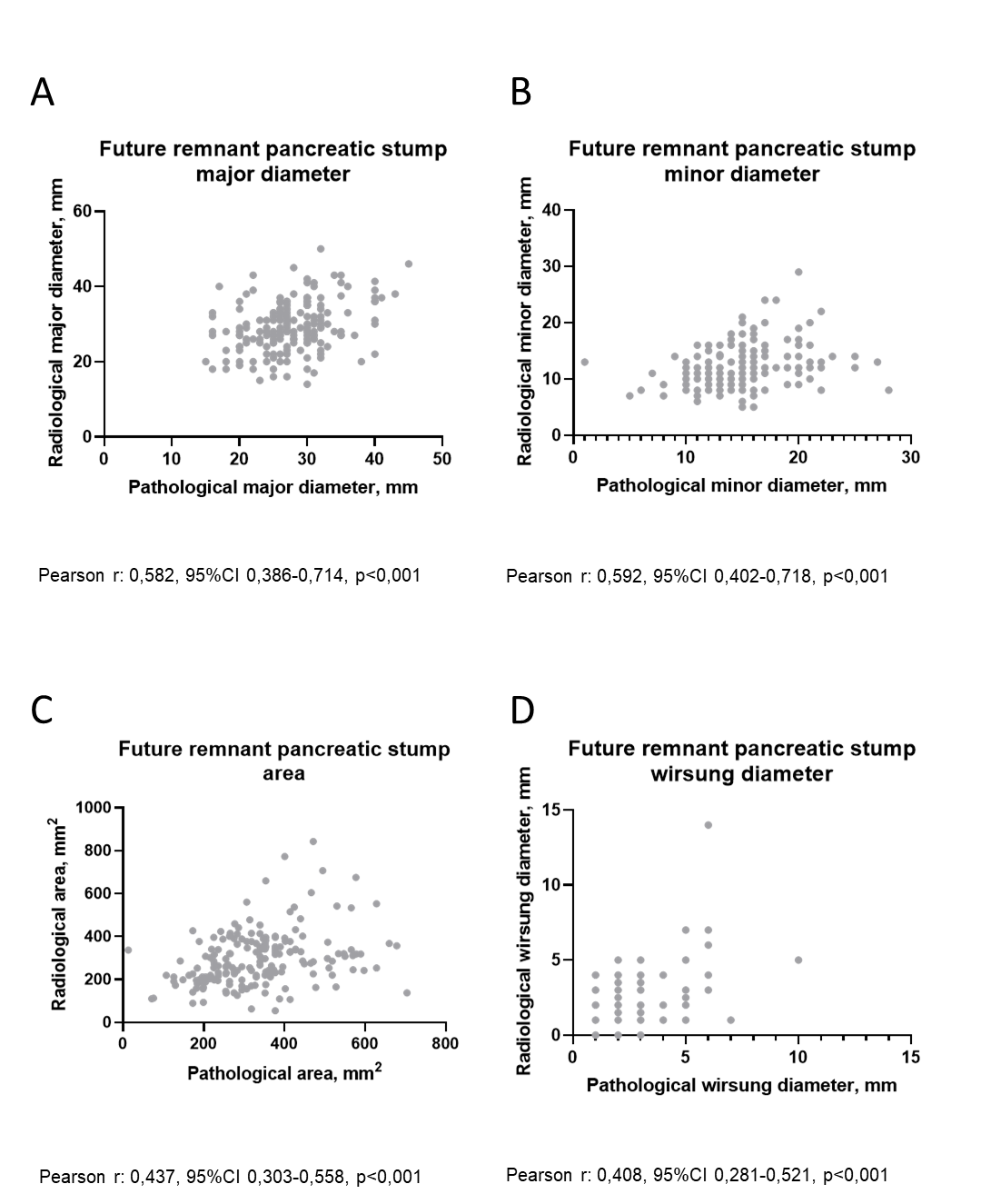
Figure S1:** Correlation between pathological and radiological pancreatic neck major diameter (A), minor diameter (B), predicted area (C) and Wirsung diameter (D).

**Figure S2:** ROC curve analysis for multivariate models B and C in the developmental cohort in predicting CR-POPF using ideal cut-offs for continuous radiological variables. AUC indicates the area under the ROC curve.

**
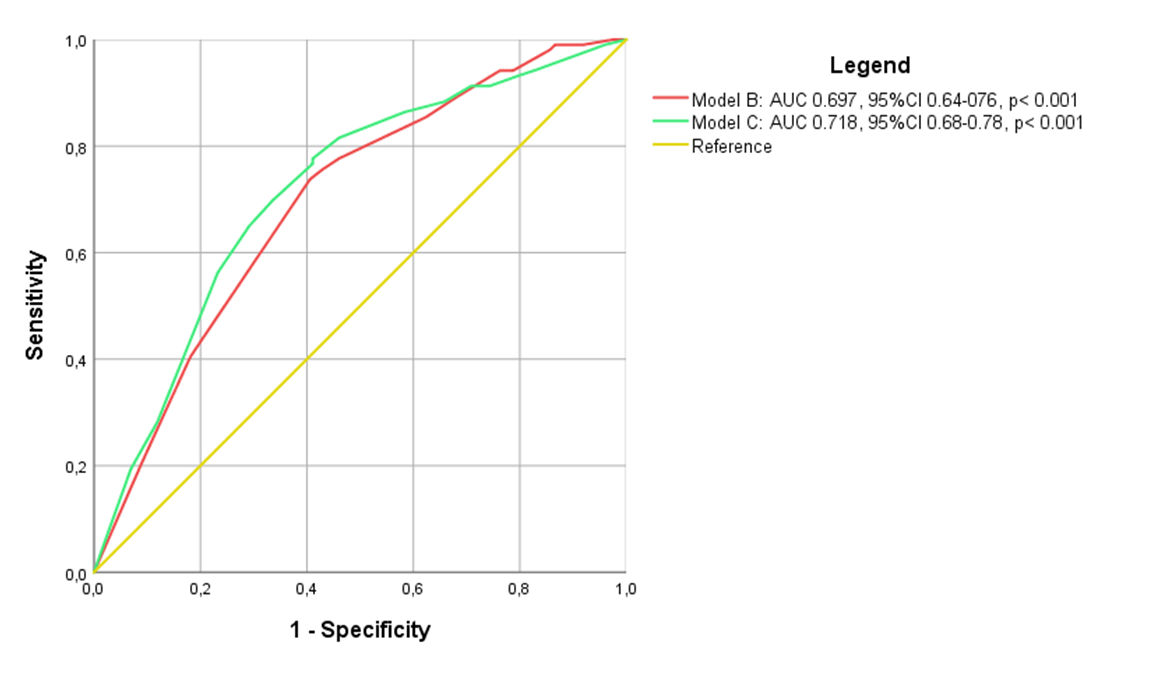
**

**Figure S3:** ROC-curve analysis for multivariate models in the developmental (A) and in the validation (B) cohorts. AUC indicates the area under the ROC-curve.


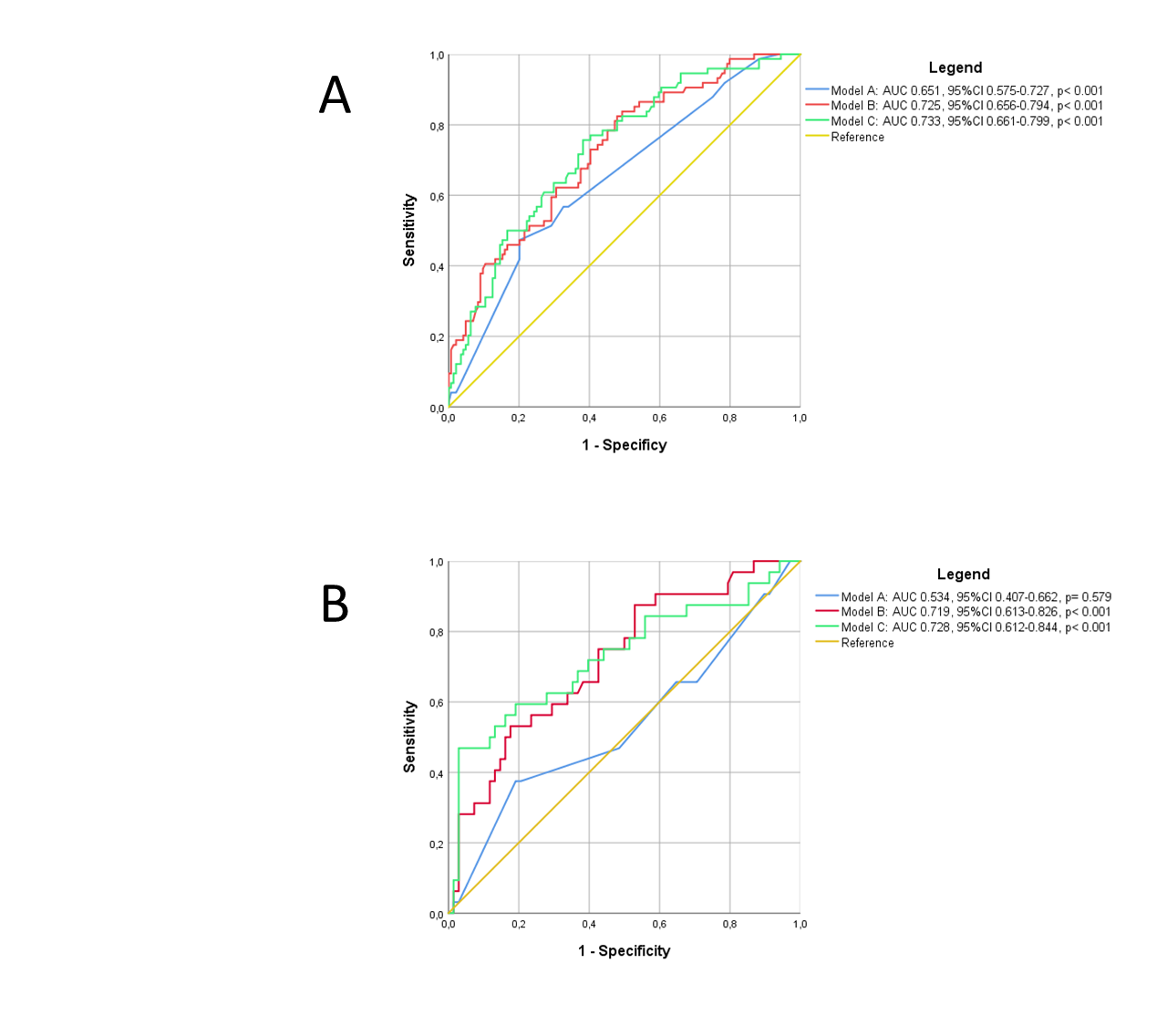


# References

1. von Elm E, Altman DG, Egger M, Pocock SJ, Gøtzsche PC, Vandenbroucke JP. The strengthening the reporting of observational studies in epidemiology (STROBE) statement: Guidelines for reporting observational studies. *Int J Surg*. 2014; **12**(12):1495-9

2. Balzano G, Guarneri G, Pecorelli N, Paiella S, Rancoita PMV, Bassi C, *et al.* Modelling centralization of pancreatic surgery in a nationwide analysis. *Br J Surg*. 2020; **107**(11):1510-1519 .

3. Pecorelli N, Mazza M, Guarneri G, Delpini R, Partelli S, Balzano G, *et al.* Impact of care pathway adherence on recovery following distal pancreatectomy within an enhanced recovery program. *HPB (Oxford)* 2021; **23**(12):1815-1823.

4. Pecorelli N, Guarneri G, Palucci M, Gozzini L, Vallorani A, Crippa S, *et al.* Early biochemical predictors of clinically relevant pancreatic fistula after distal pancreatectomy: a role for serum amylase and C-reactive protein. *Surg Endosc* 2022; doi:10.1007/S00464-021-08883-3 [Epub ahead of print].

5. Utsumi M, Aoki H, Yabuki T, Nagahisa S, Nishimura S, Une Y, *et al.* The Late Phase/Early Phase Ratio of Pancreatic CT Values as a Novel Predictor of Pancreatic Fistula after Distal Pancreatectomy. *Acta Med Okayama* 2020; **74**: 351–358.

6. Tanaka K, Yamada S, Sonohara F, Takami H, Hayashi M, Kanda M, *et al.* Pancreatic Fat and Body Composition Measurements by Computed Tomography are Associated with Pancreatic Fistula After Pancreatectomy. *Ann Surg Oncol* 2021; **28**: 530–538.

7. Pecorelli N, Carrara G, De Cobelli F, Cristel G, Damascelli A, Balzano G, *et al.* Effect of sarcopenia and visceral obesity on mortality and pancreatic fistula following pancreatic cancer surgery. *Br J Surg* 2016; **103**: 434–442.

8. https://www.tomovision.com/SliceO_Help/index.htm?context=930 [Internet]. [cited 2022 Jan 10]. Available from: https://www.tomovision.com/SliceO_Help/index.htm?context=930

9. Bassi C, Marchegiani G, Dervenis C, Sarr M, Abu Hilal M, Adham M, *et al.* The 2016 update of the International Study Group (ISGPS) definition and grading of postoperative pancreatic fistula: 11 Years After. *Surgery* 2017; **161**: 584–591.

10. Wente MN, Bassi C, Dervenis C, Fingerhut A, Gouma DJ, Izbicki JR, *et al.* Delayed gastric emptying (DGE) after pancreatic surgery: A suggested definition by the International Study Group of Pancreatic Surgery (ISGPS). *Surgery*. 2007; **142**(5):761-768.

11. Wente MN, Veit JA, Bassi C, Dervenis C, Fingerhut A, Gouma DJ, *et al.* Postpancreatectomy hemorrhage (PPH)-An International Study Group of Pancreatic Surgery (ISGPS) definition. *Surgery*. 2007; **142**(1):20-25.

12. Dindo D, Demartines N, Clavien PA. Classification of surgical complications: A new proposal with evaluation in a cohort of 6336 patients and results of a survey. Ann. Surg. 2004; **240**(2):205-13.

13. Slankamenac K, Graf R, Barkun J, Puhan MA, Clavien PA. The comprehensive complication index: A novel continuous scale to measure surgical morbidity. *Ann Surg*. 2013; **258**(1):1-7.

14. Landis JR, Koch GG. The measurement of observer agreement for categorical data. *Biometrics* 1977; **33**: 159.

15. Schober P, Schwarte LA. Correlation Coefficients: Appropriate Use and Interpretation. *Anesth Analg* 2018; **126**: 1763–1768.
